# IL-17 A correlates with disease progression in papillary thyroid carcinoma

**DOI:** 10.1186/s13000-023-01362-4

**Published:** 2023-08-10

**Authors:** Sohini Banerjee, Uma Nahar, Divya Dahiya, Rijuneeta Gupta, Soham Mukherjee, Naresh Sachdeva, Ashwani Sood, Pranab Dey, Bishan Radotra, Anil Bhansali

**Affiliations:** 1 Department of Histopathology, Post Graduate Institute of Medical Education and Research Chandigarh, 160012 Chandigarh, India; 2 Department of General Surgery, Post Graduate Institute of Medical Education and Research Chandigarh, 160012 Chandigarh, India; 3 Department of Otolaryngology (ENT), Post Graduate Institute of Medical Education and Research Chandigarh, 160012 Chandigarh, India; 4 Department of Endocrinology, Post Graduate Institute of Medical Education and Research Chandigarh, 160012 Chandigarh, India; 5 Department of Nuclear Medicine, Post Graduate Institute of Medical Education and Research, Chandigarh, 160012 India; 6 Department of Cytology and Gynaecological Pathology, Post Graduate Institute of Medical Education and Research Chandigarh, 160012 Chandigarh, India

**Keywords:** IL-17A, Papillary thyroid carcinoma, Disease progression, FoxP3

## Abstract

**Background:**

Cancer progression can be promoted by chronic inflammation. Local immune response may be associated with favourable or unfavourable prognosis of Papillary Thyroid Carcinoma (PTC). Regulatory T (Treg) cells and T helper 17 (Th17) cells exert opposing function and their balance may have a vital role in promotion of tumor growth. Treg cells in tumor microenvironment (TME) may promote tumor progression and reduced survival of patients. Whereas, Th17 cells can promote or inhibit tumor progression depending on phenotypic characteristics of tumor. In this study, we aimed to analyse the kind of immune response developed and its prognostic impact in future therapeutics.

**Methods:**

Cytometric Bead Array (CBA) analysis of pro and anti-inflammatory cytokines (IFN-gamma, IL-2, IL-6, IL-17 A, TNF-alpha and IL-4, IL-10) was done in 15 PTC irrespective of Lymphocytic Thyroiditis (LT) and 16 Hashimoto’s Thyroiditis (HT) cases. Immunohistochemical expression of FoxP3 and IL-17 A was studied in 27 cases of PTC with LT. Whereas, quantitative gene expression of both was analysed in 10 cases.

**Results:**

All the pro-inflammatory cytokines showed mild elevation in PTC with LT. On IHC, IL-17 A expression was observed in 74% PTC with LT. Whereas, FoxP3 was present in only 40% cases. Also, IL-17 A expression was significantly associated with age group (> 45 years), tumor size ≤ 1 cm and disease progression.

**Conclusions:**

Increased expression of cytokines suggested correlation between inflammatory factors and progression of thyroid tumors. Along with this, the balance between IL-17 A and FoxP3 may play an important role in PTC development, prognosis and future management.

## Background

Thyroid cancer accounts for around 2% of all human cancers. Most of these patients respond well to conventional therapy; however, 10–30% of them may present with recurrent disease [1]. Some may eventually stop responding to radioiodine treatment and metastasize to a distant part of the body. A mixture of immune cells is found within or even surrounding primary thyroid tumors, indicating active involvement of the immune system in papillary thyroid carcinoma (PTC) [2]. Pathologists often recognize cells’ infiltration from innate and adaptive immune systems, which may indicate inflammatory conditions in non-neoplastic tissues.

Further, clinicians have long been realizing local immune response, and concurrent Chronic Lymphocytic Thyroiditis (CLT) can be associated with a favorable or unfavorable prognostic profile of PTC patients [3, 4]. CLT prevalence in patients with PTC has been reported to be significantly higher than with benign thyroid tumors [5]. Moreover, patients with CLT remain at higher risk for PTC compared with patients without CLT. The reason behind is that inflammatory mediators in tumor microenvironment (TME) can promote tumor progression by remodeling tumor stroma and stimulating angiogenesis. In contrast, tumor cells may secrete molecule which create an immunosuppressive microenvironment which followed by the recruitment of regulatory T lymphocytes [6, 7]. However, the nature of this lymphocytic reaction is not well understood. In highly immunogenic tumors, spontaneous protective immunity can be generated against tumors; whereas in poorly immunogenic tumors, functional T cell response usually is not generated. In highly immunogenic tumors, interleukins (ILs) as a component of TME plays a significant role in tumor progression. They often act as pro-angiogenic factors which further may develop an antitumor response in these cases, but it does not control tumor progression rather than favor tumor growth. Tumor necrosis factor alpha (TNF-α), un this context, has been shown to have dual role on tumor development. On one hand, TNF-α may promotes angiogenesis and metastasis of tumor cells at low concentrations, and at high levels it may exert antitumor effects [8]. Additionally, interleukin-2 (IL-2), a multifunctional cytokine is known to activate T cells in TME of thyroid cancer by upregulating Human Leukocyte antigen (HLA) class I molecule expression and subsequent tumor antigen presentation [9].

A large proportion of cancers represent this kind of immune response, including Thyroid carcinoma (TC). The presence of intratumor or peritumoral infiltration of lymphocytes is evidence that the immune system may respond to malignant transformation as previous studies have shown dense CD8 + T cells in cancer cell nests may be correlated with prognosis. A 9-year follow-up study revealed that PTC with tumor-infiltrating lymphocytes (TILs) were less prone to tumor recurrence than PTC without TILs. This suggests that TILs may predict a favorable prognosis in these patients. However, lymphocytes are a pool of cells in which multiple phenotypes can be found [10]. Therefore, it is essential to assess different subsets of TIL to study prognostic prediction. CD4 + T cells play a key role in regulating immune response generated in malignant transformation. It can be differentiated into one of at least four functionally distinct populations of cells: T helper 1 (Th1), Th2, regulatory T (Tregs), or Th17. Th1 polarization can be characterized by interferon (IFN) -𝛾 production and may support the cytotoxic response mediated by CD8 + T cells [11].

Conversely, Th2 polarization can stimulate humoral immunity combined with suppressed cellular immunity and failed cytotoxic T-cell antitumor immunity by altering cancer risk toward tumor promotion and progression. Treg is generally identified as FoxP3 + lymphocytes [12, 13], induced forms of thought to contribute to tumor-specific T-cell tolerance and suppress autoimmunity and antitumor immunity through secretion of inhibitory cytokines such as IL-10 and transforming growth factor-beta (TGF-β) [14]. Conversely, the recently discovered Th17 cells play an essential role in promoting autoimmunity, carcinogenesis, and antitumor immunity. In this present study, we wanted to observe the imbalance in Treg and Th17 cells’ expression in terms of analyzing the transcription factor FoxP3 and the secretory cytokine IL-17 A, respectively, to explore the regulation of antitumor immune response generated in tissues of PTC.

## Methods

### Patients and samples

**Fig. 1:**
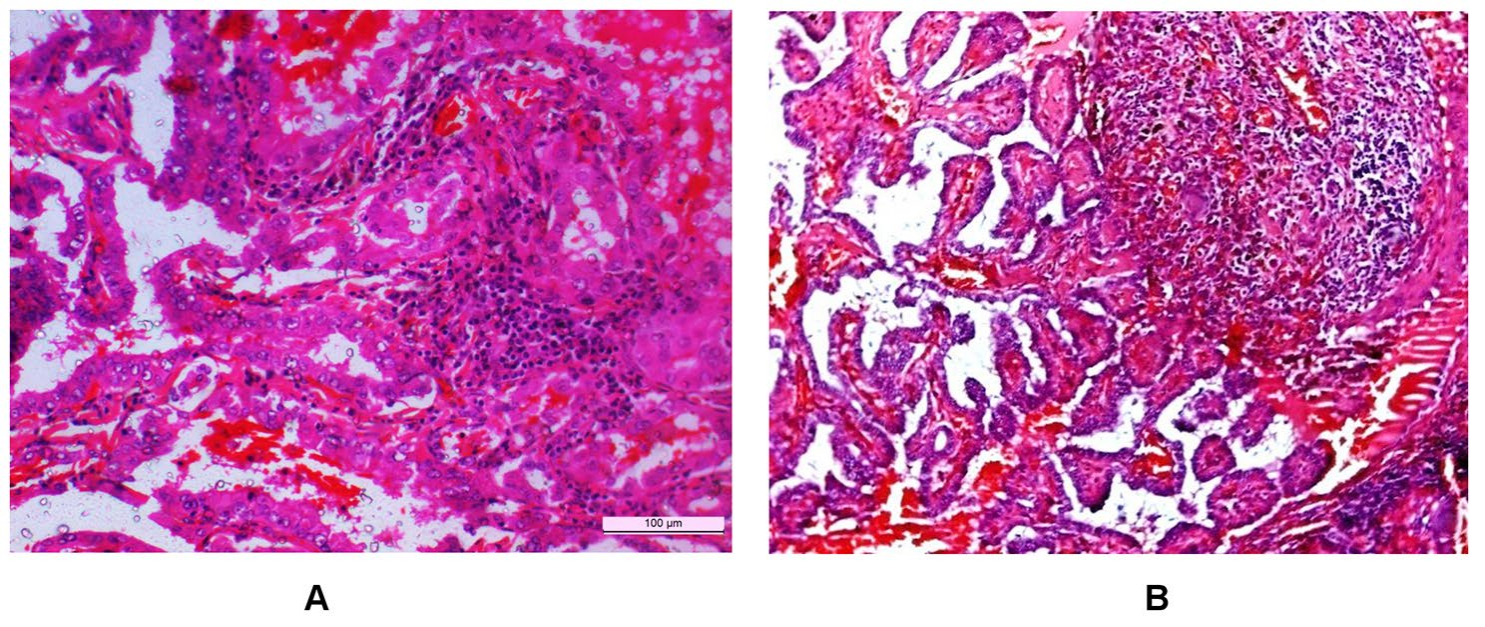
Photomicrographs showing papillary thyroid carcinoma (**A**) with tumor infiltrating lymphocytes and (**B**) associated with lymphocytic thyroiditis (H&E 20X)

The study was conducted at Post Graduate Institute of Medical Education and Research (PGIMER) Chandigarh, India from January 2015 to December 2019. Pre-operative peripheral blood samples from consecutive PTC with LT and benign hyperplastic nodules (HN) with LT cases were collected for Cytometric Bead Array (CBA) analysis. Histopathologically proven PTC with LT and HN with LT cases of both sex with age above 18 years were included for immunohistochemical evaluation (Fig. 1). Hashimoto’s Thyroiditis patients were enrolled from the Department of Endocrinology. The fresh tumor tissues were collected at the time of surgery, stored in a sterile condition and transferred to RNA Later for subsequent RNA extraction.

An informed consent from all the patients was collected. The ethical clearance was obtained from the institute’s ethics committee. The demographic and clinical details of PTC patients including age, sex, tumor stage and lymph node metastasis were retrieved from histopathology report and clinical records from the department of Histopathology and Endocrinology respectively. The histologic subtype was confirmed by reviewing Haematoxylin and Eosin (H&E) slides unbiaslly by two observers (UN and SB).

### Cells and serum

5 ml of Venous blood was collected in vacutainers from all the patients enrolled in this study. For serum isolation, blood samples were centrifuged at 2300 rpm for 10 min, without brake. The serum separated, collected carefully in a 1.5 ml microcentrifuge tube, and stored in -80ºC deep freezer for further analysis.

### Cytometric bead array

Serum samples isolated from peripheral blood of PTC with LT patients were assayed for pro-inflammatory (IFN-γ, IL-2, IL-6, IL-17 A and TNF-alpha) and anti-inflammatory (IL-4, 10) cytokines using manufacture’s protocol of BD Th1/Th2/Th17 Cytometric Bead Array kit (cat no. 560,484) in comparison to Hashimoto’s Thyroiditis patients. Cytokine levels were measured using corresponding cytokine standards [15].

### Immunohistochemistry (IHC)

The formalin-fixed, paraffin-embedded (FFPE) tissue blocks were used for IHC using standard protocol. Antigen retrieval was done by dipping the slides in Sodium Citrate buffer (pH 6) for 10 min in a decloaking chamber, and the slides were removed after full pressure release. The slides were cooled, followed by endogenous peroxide blocking by incubation with 3% H2O2 for 30–45 min. The sections were washed with 1X PBST and then incubated with the primary antibody to human FoxP3 (Abcam, diluted 1:75) and IL-17 A (R&D, diluted 1:50) at room temperature for 45–60 min. The slides were washed with 1X PBST, and a secondary antibody was added, followed by DAB and haematoxylin counterstaining.

### Immunohistochemical scoring

The immunoreactivity of Foxp3 and IL-17 A was scored semiquantitatively.

The intensity was graded as 0 (negative), + (weak), ++ (moderate) and+++ (strong). The frequency was graded from 0 to 4 by percentage of positive cells:

- grade 0:<3% of positive cells.
- grade 1: 3–25%.
- grade 2: 26–50%.
- grade 3: 51–75% and.
- grade 4:> 75% of positive cells [16].

The index score was calculated by multiplying the intensity and frequency grades, which was further placed into a 4-point scale:

- index score 0 (product of intensity and frequency is 0).
- 1 (+, the product is 1 and 2).
- 2 (++, the product is 3 and 4).
- 3 (+++, the product is in between 6 and 12).

### RNA extraction and cDNA synthesis

Total RNA was extracted from the fresh tissues of PTC collected at the time of surgery using the miRvana Paris Kit according to the manufacturer’s instructions (Invitrogen) and quantified spectrophotometrically; its integrity was verified by 1% agarose gel electrophoresis. The spectrophotometer reading for RNA of all the samples included was in between 1.9 and 2.0 and agarose gel electrophoresis showed mostly 3 bands and in some samples 2 bands for RNA samples. We reverse-transcribed one ug of total RNA in a total volume of 20 ul using a cDNA synthesis kit (Bio-Rad). cDNA was stored in a -20^o^C refrigerator for further use in quantitative RT-PCR [17].

### Quantitative real-time RT-PCR

Copy numbers of FoxP3 and IL-17 A mRNA was determined by quantitative real-time RT-PCR using a Light cycler (Applied Biosystems). Master SYBR Green (Applied Biosystems) was used for the gene analysis. Amplifications (10 ul reaction volume) were performed in 96 well plates; 1 ul of cDNA, 5 ul of SYBR Green, 4 ul of sterile water, and 0.5 ul of each primer were used. The RT-PCR reactions were performed according to the Light cycler standard protocol using the annealing temperature of 66 °C for FoxP3 and 63.8ºC for IL-17 A. Cycle threshold (C_T_) values and calculated values were determined using the Light cycler machine. Samples were loaded in duplicate, and the data was calculated in terms of mean. Mastermix, with the addition of 1 ul of sterile water instead of cDNA, served as a negative control. Delta delta Ct method was used to calculate the fold changes (expression level) of target genes in comparison to reference gene. GAPDH was used as reference gene in these samples.

### Statistical analyses

Statistical analysis was performed using Graph Pad Prism Software. Data were tested for normal distribution using Shapiro-Wilk test. Data were presented as mean ± SD where normally distributed and as median (interquartile range) where data were skewed. The data were analysed by 2 × 2 contingency table and Chi-square test. The differences were considered significant where the p value was < 0.05.

## Results

A total of 27 cases of PTC with LT and 12 benign thyroid nodule cases were enrolled in the study after histological confirmation. There were 20 females and only 7 males with male: female ratio of 1: 2.8. The clinical details are depicted in Table 1. The overall survival was 88% after a mean follow up of 30.33 ± 4.67 months. Three out of those survived (n = 16) had shown disease progression during the follow-up period.

**Table 1:** Clinical details of patients

Parameters	PTC with LT (n = 27)
Mean age	36.19 ± 2.43*
Mean tumor diameter (cm)	2.4 ± 0.3*
Histological subtype	Classical 96% (26/27) Follicular variant 4% (1/27)
Tumor diameter ≤ 2 cm > 2 to ≤ 4 cm > 4 cm	54% (13/24) 37.5% (9/24) 8.5% (2/24)
Cervical LNM	52% (13/25)
Mean follow-up (months)	30.33 ± 4.67*
Distant metastasis	27.8% (5/18)
Mortality	11.1% (2/18)

Data represented in * Mean ± SEM.

### Serum cytokine detection in PTC with LT

**Fig. 2:**
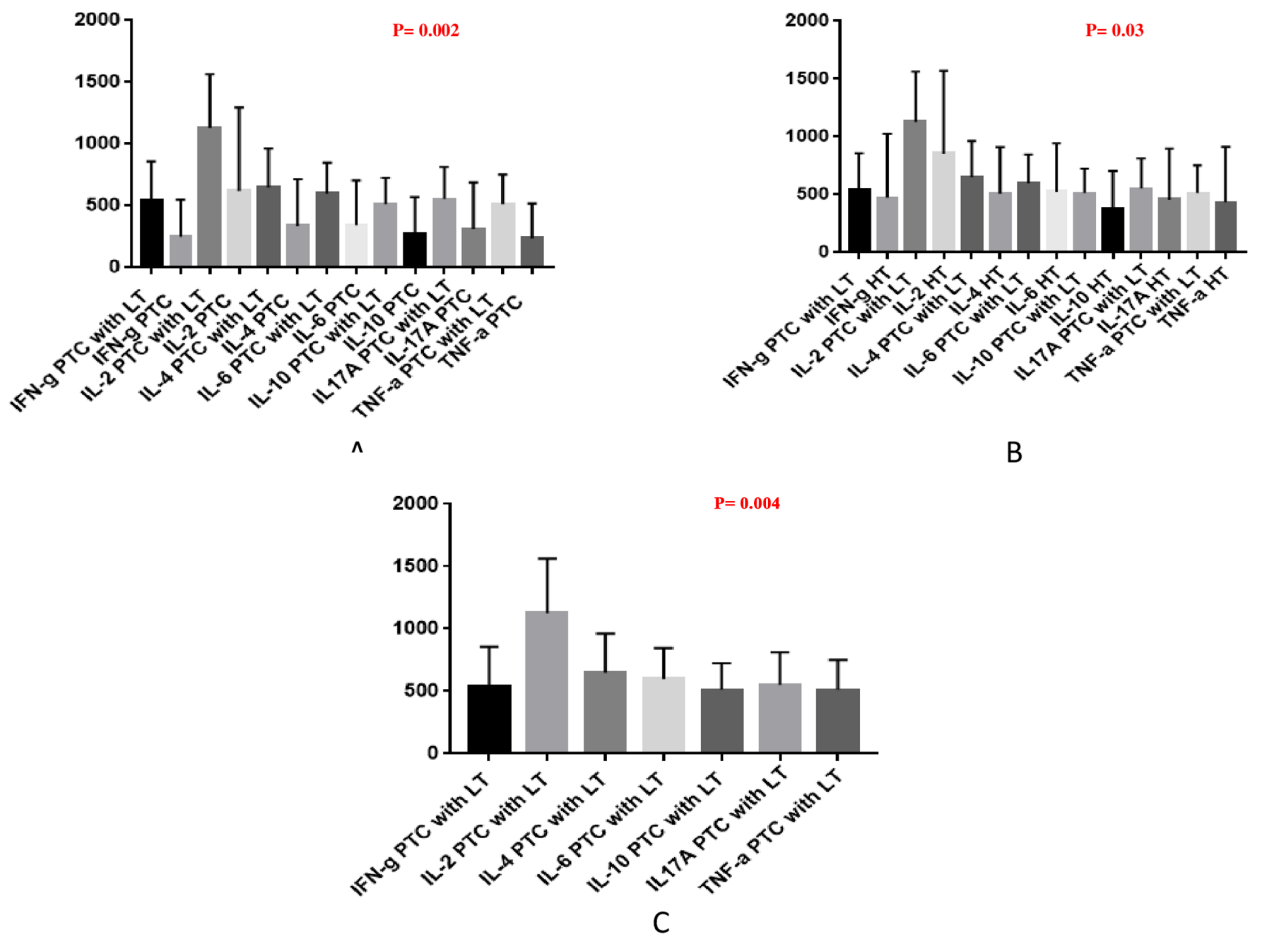
(**A-C**): Serum cytokine concentration in PTC with LT

CBA was done by Th1/Th2/Th17 Cytokine kit as per the manufacture’s protocol in Hashimoto’s thyroiditis (n = 16) and Papillary Ca thyroid irrespective of LT (n = 15). The level of cytokines measured included IFN-g, IL-2, IL-6, IL-17 A, TNF-a, IL-4, IL-10 (in comparison to standards provided with Kit). The results are shown in Fig. 2 (A to C). The median level of all the pro-inflammatory cytokines (IFN-g, IL-2, IL-6, IL-17 A, TNF-a) cytokines in PTC with LT patients (n = 7) showed elevation to that of PTC only (n = 8) (P = 0.002) and HT (n = 16) (P = 0.03). Further, on comparing median level of the cytokines in the same group of PTC with LT, we observed IL-2 was the most significantly up regulated than other pro-inflammatory cytokines (P = 0.004).

### Characterization of Tregs and Th17 cells in PTC with LT by IHC

**Fig. 3:**
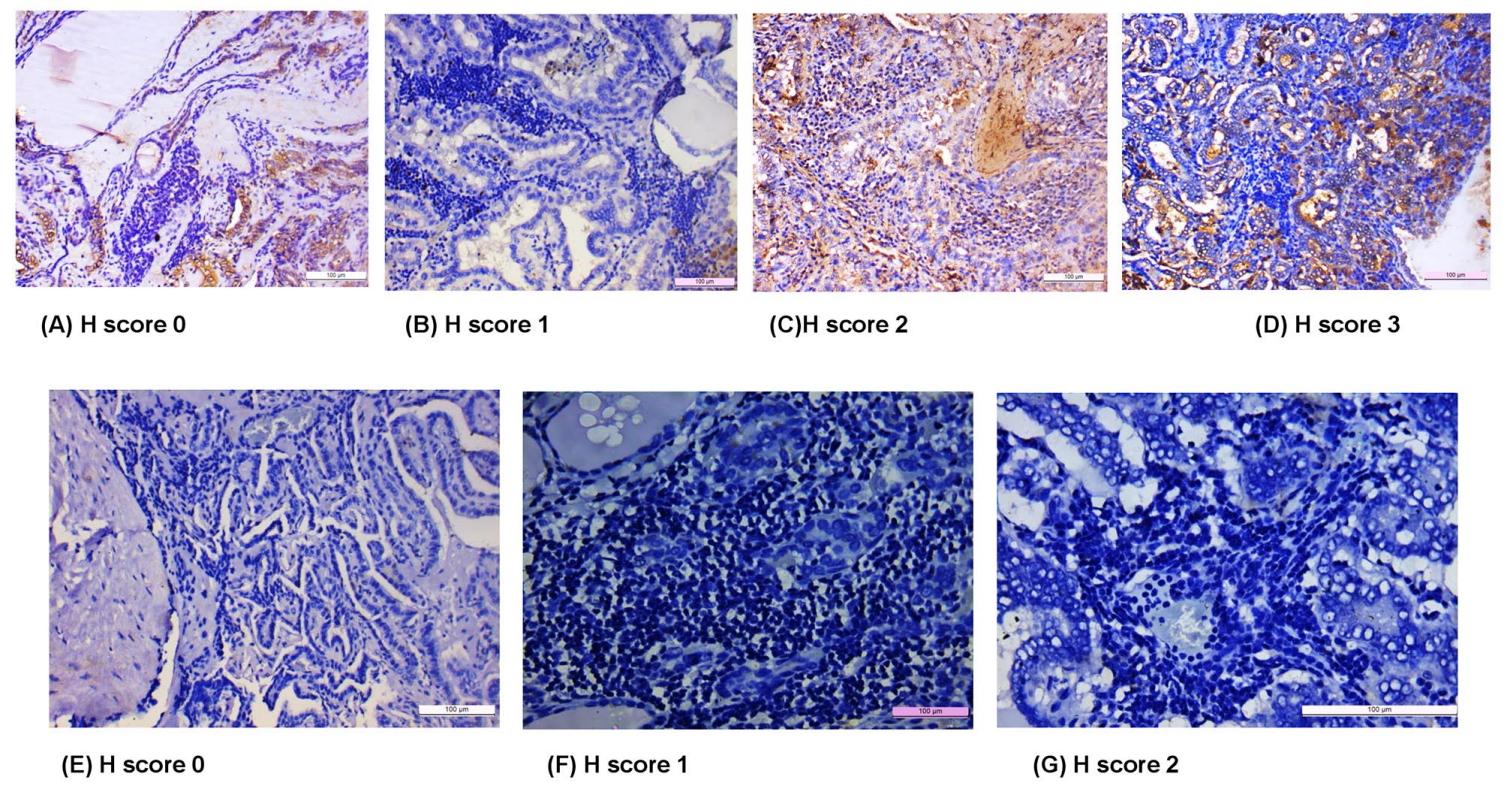
(**A-D**) Expression of Treg cells, (**E-G**) Expression of Th17 cells

**Fig. 4:**
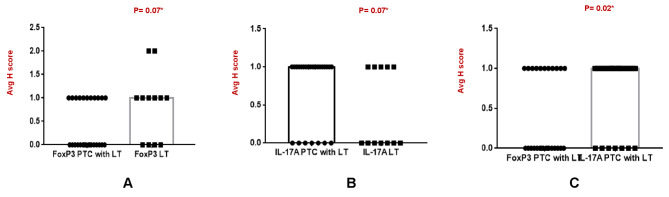
(**A-C**) Relative protein expression of FoxP3 & IL-17A

The IHC for FoxP3 (transcription factor of Treg cells) and IL-17 A (cytokine secreted from Th17 cells) was done in 27 samples of PTC with LT (n = 27) included in this study. Of the total 27 cases, FoxP3 showed nuclear positivity in 40% cases of PTC with LT with an intensity varied from 1 + to 3+ (Fig. 3A and D). In comparison, 67% of LT cases showed FoxP3 positivity with an intensity varied between 1 + and 2+ (Fig. 4A). Therefore, our results showed a decreased expression of FoxP3 in PTC with LT cases compared to benign LT though it was statistically significant (P = 0.07). On the contrary, IL-17 A showed 74% positivity in PTC with LT, where the intensity varied between 1 + and 2+ (Fig. 3E and G). In comparison, benign LT showed 42% positivity for IL-17 A with an intensity of 1 + only (Fig. 4B). Hence, significantly increased expression of IL-17 A was seen in PTC with LT compared to LT cases (P = 0.07). Further, IL-17 A expression also showed significant increase in comparison to FoxP3 expression in PTC with LT cases (P = 0.02) (Fig. 4C).

### mRNA expression of Treg and Th17 cells in PTC with LT in comparison to benign LT by Real-Time quantitative PCR

**Fig. 5:**
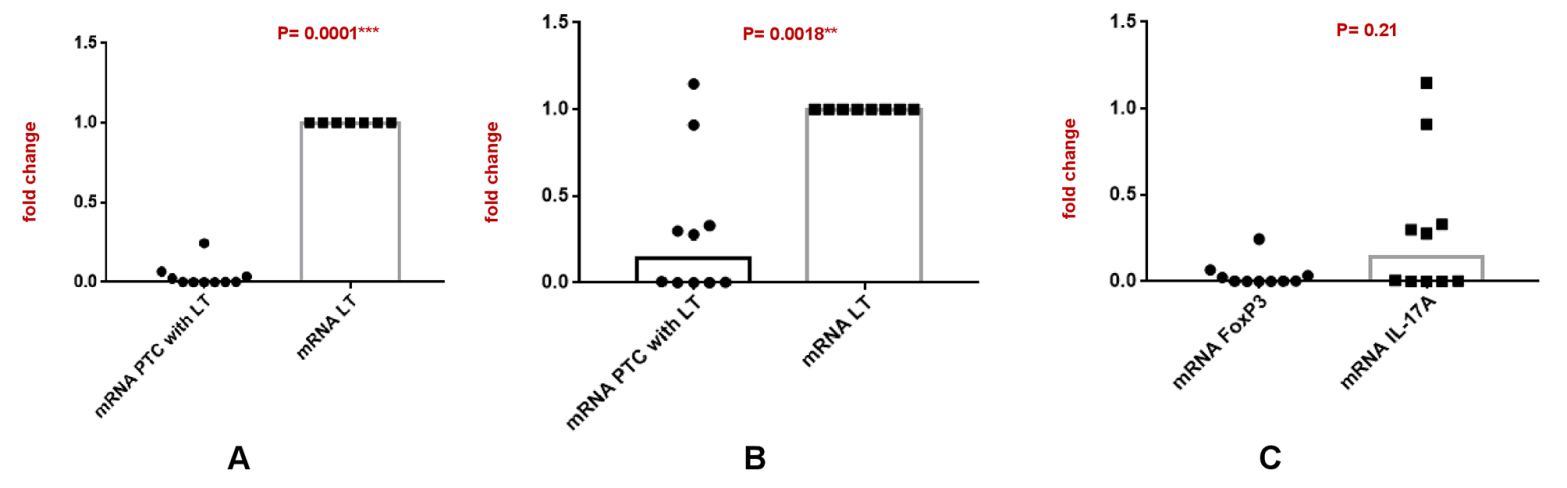
(**A-C**) Relative mRNA expression of FoxP3 & IL-17A

Quantitative real-time PCR analysis of FoxP3 and IL-17 A was done in 10 cases of PTC with LT cases. We observed significant downregulation of FoxP3 mRNA (Fig. 5A, P = 0.0001***), and IL-17 A mRNA (Fig. 5B, P = 0.0018**) in PTC with LT compared to LT. We also analyzed the balance in the expression level of FoxP3 and IL-17 A (Fig. 5C) in PTC with LT. Here we noticed mild increase in expression of IL-17 A concerning FoxP3 (P = 0.21) with an upward trend in PTC with LT cases. All the graphs are shown in Fig. 5 (A-C).

### Clinicopathological correlation of IL-17 A protein expression

**Fig. 6:**
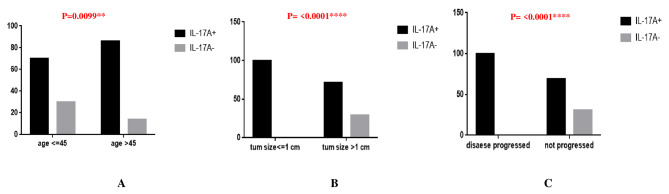
(**A-C**): IL-17 A & its clinicopathological correlation

In this study, we observed significant association in between IL-17 A expression and patients’ age > 45 years, even smaller tumor size (≤ 1 cm) and disease progression in follow up period (Fig. 6A-C; Table 2). However, we did not find any significant correlation between Foxp3 + Tregs expression and clinical parameters of patients.

**Table 2:** Association between IL-17 A expression and clinicopathological parameters

Parameters	IL-17 A+ (20/27 = 74%)	IL-17 A- (7/27 = 26%)	P value
age ≤ 45	70	30	**0.0099**
age > 45	86	14	
tum size ≤ 1 cm	100	0	**< 0.0001**
tum size > 1 cm	71	29	
multifocality +	83	17	0.1239
multifocality -	73	27	
LNM +	77	23	0.1560
LNM -	67	33	
disease progressed	100	0	**< 0.0001**
not progressed	69	31	

## Discussions

There is a functional relationship between chronic immune responses and cancer which is not clearly understood in PTC. Chronic inflammation can often be correlated with carcinogenesis through the relative contribution of two specific lymphocyte subsets, i.e., Treg cells and Th17 cells [18]. However, in PTC, limited literature on balance between these two cell types is available. We analysed the expression of these two cells in PTC tissue. We measured the level of pro and anti-inflammatory cytokines, namely IFN-g, IL-2, IL-6, IL-17 A, TNF-a, IL-4, IL-10 in serum of PTC cases irrespective of LT and compared to HT.

Serum level of all pro and anti-inflammatory cytokines IFN-g, IL-2, IL-6, IL-17 A, TNF-a, IL-4, IL-10 was detected in PTC with LT, PTC only and HT. Our result showed significantly increased expression of all the pro (IFN-g, IL-2, IL-6, IL-17 A, TNF-a) and anti-inflammatory cytokines (IL-4 and IL-10) in PTC with LT compared to PTC only and HT. Of all the cytokines, IL-2 was the most upregulated, followed by IL-4, IL-6 and IL-17 A, whereas, IL-10 was the least elevated one observed in our study. Therefore, pro-inflammatory mediators were more prevalent in the systemic circulation of PTC with LT patients. Previously, HU et al. has observed increased IL-2 expression in PTC + HT tissues. They observed that IL-2 treated PTC cells can limit tumor progression by increasing activation of CD8 + T cells and downregulation of immune checkpoint inhibitor PD-1. Their findings suggested IL-2 as a promising immunotherapy in PTC [9]. The second most upregulated cytokine was IL-4 in our study. It was elevated in two of our PTC with LT patients who had progressed the disease in the follow-up period. Our observation supported the previous study by Stanciu et al., which suggested its need for close monitoring and intensive treatment of PTC. Similarly, Simonovic et al. also found elevated IL-4 in peripheral blood of PTC with autoimmune HT patients. Also, Stassi et al. detected the role of IL-4 in inhibiting apoptosis by B and T cells through recruiting anti-apoptotic molecules [19–21]. Another cytokine IL-6 was also elevated in our study, especially in two patients with disease progression in follow up period. Our observation was concordant with Kobawala et al. who have noticed significant association of IL-6 level and poor survival of PTC patients [22]. Together, all these cytokines may activate different cell signaling pathways to promote angiogenesis, regulate immune response in TME, inhibit tumor cell apoptosis and further distant metastasis followed by poor overall survival. In this context, IL-17 A cytokine is the mostly known as pro-angiogenic mediator now a days, due to its involvement in inflammatory process and TNF-alpha secretion in tumor microenvironment [23, 24]. Therefore, we studied both local and systemic expression of IL-17 A in PTC with LT patients. IL-17 A level was elevated in serum of PTC with LT patients. Also, tumor size of all these patients were ≥ 1 cm and 43% of these were presented with lymph node metastasis. Similar to our study, Jiang et al. correlated IL-17 expression with TNM stage, and lymph node metastasis of thyroid cancer. Again, Carvalho et al. correlated serum IL-17 level with shorter recurrence free survival in thyroid cancer patients [25, 26]. All of these pro-inflammatory cytokines were associated with certain parameters of PTC tumorigenesis and progression of the disease. On the other hand, the anti-inflammatory cytokine IL-10 was known to influence PTC’s aggressiveness by inhibiting immune cells’ anti-tumor activity [27]. However, in our study we observed IL-10 cytokine was less elevated with respect to other cytokines.

Immunohistochemical expression showed that FoxP3 + Treg cells were less frequent in PTC with LT than in benign LT. Only 40% (11/27) PTC with LT cases were positive with mild to moderate nuclear expression of FoxP3. Similarly, Mohamed et al. reported 45% positivity of FoxP3 followed by Ugolini et al. who also reported 43% positivity of FoxP3 in PTC cases [28, 29]. In contrast, Cunha et al. found 91.9% positivity of FoxP3 at cytoplasm and nucleus in PTC compared to nodular goitres. They correlated FoxP3 nuclear expression to the aggressiveness of differentiated thyroid carcinomas. Additionally, FoxP3 + lymphocytic infiltration was more frequent in PTCs smaller than 2 cm in diameter, lacking extrathyroidal invasion and associated with chronic lymphocytic thyroiditis [30].

On the other hand, significantly upregulated expression of IL-17 A was seen in PTC with LT compared to benign LT, and the expression varied between mild and moderate intensity. In our study, 74% (20/27) of cells were positive for IL 17 in all papillary thyroid tumors associated with lymphocytes surrounding the lesions. Similarly, Carvalho et al. reported higher IL-17 expression in differentiated thyroid carcinoma than that of benign thyroid neoplasms and its association with high recurrence and morality as a pro-angiogenic mediator [26]. Han et al. identified an elevated IL-17 A expression in PTC with coexisting HT. Also, they reported that administration of IL-17 A can effectively induce Major Histocompatibility complex I (MHC class I) expression in PTC cell lines (namely K1 and TPC-1) in vitro and subsequent reduction of PD-1 expression. The PD-1 expression in PTC may exhaust the effector T cells to avoid immune attack and killing of tumor cells [31]. Therefore, its reduction led to increased T cell activation with IL-2 cytokine production and subsequent suppression of tumor immune escape by inhibiting PD-1/PD-L1 pathway [32]. Further, protein expression showed significant up regulation of IL-17 A secreting Th17 cells compared to FoxP3 expressing Treg cells in IHC (P = 0.02). Earlier Jeon et al. have demonstrated that IL 17 can induce vascular endothelial growth factor expression with subsequent secretion of TGF β and high levels of TGF β further promoted tumor growth and metastasis [33]. Kryczek et al. also observed Th17 cells correlating with immune effector cells, including CD8 + T cells and natural killer cells and thus promoting an antitumor response mediated by cytotoxic cells [34].

To validate the results, quantitative real time PCR revealed significant downregulation of both FoxP3 and IL-17 A mRNA in PTC with LT compared to benign HN with LT. However, IL-17 A mRNA was significantly upregulated compared to FoxP3 mRNA in PTC with LT cases.

In this study, we observed significant association between IL-17 A expression and patients’ age > 45 years, even smaller tumor size (≤ 1 cm), and disease progression in the follow-up period. However, we did not find any significant correlation between Foxp3 + Tregs expression and the clinical parameters of patients. Previously, high IL 17 protein expression was associated with recurrence in around 74% of patients who succumbed to the disease. This indicated that IL 17 cytokine may be involved in the development and progression of tumors and can present a prognostic factor for poor survival [35]. Zhang et al. have demonstrated that the accumulation of IL 17 producing cells in the tumor microenvironment may lead to tumor progression in patients with hepatocellular carcinoma by promoting angiogenesis [36].

## Conclusions

Our study detected a lower expression of FoxP3 + regulatory T cells in PTC with LT patients. Also, IL-10 cytokine secretion was more deficient in these patients’ peripheral blood. On the other hand, we observed increased expression of IL-17 A secreting Th17 cells in PTC with LT patients. Also, the median level of IL-17 A cytokine was upregulated in peripheral blood of these patients. At the gene level, IL-17 A mRNA showed an upward trend compared to FoxP3 mRNA. Finally, IL-17 A expression was significantly associated with even smaller tumor diameter and disease progression which reflects its significance as an early diagnostic and future prognostic marker of PTC. These results suggested that infiltration of IL-17 A + cells in the tumor microenvironment of PTC with LT possibly helping tumors to sustain the growth and development by inhibiting antitumor immune responses and Tregs, in this context, playing comparatively non-significant role in concurrent PTC with LT by having comparatively less elevated IL-10 secretion.

## Data Availability

The datasets used and/or analysed during the current study are available from the corresponding author on reasonable request.

## List of Abbreviations

PTC: Papillary thyroid carcinoma
LT: Lymphocytic thyroiditis
HT: Hashimoto’s thyroiditis
Treg: T regulatory cell
Th17: T helper 17
TME: Tumor microenvironment
CBA: Cytometric bead array
ILs: Interleukins
